# Distribution characteristics and clinical correlation of inhalant allergen sensitization in children with respiratory and allergic diseases

**DOI:** 10.1097/MD.0000000000048087

**Published:** 2026-03-13

**Authors:** Wei Wang, Qingling Liu, Qianqian Zhang, Dongqing Ma, Hong Wang

**Affiliations:** a Department of Pediatrics, Civil aviation General Hospital, Beijing City, China.

**Keywords:** allergic rhinitis, asthma, inhalant allergen, pediatric respiratory allergic diseases, specific immunoglobulin E, total immunoglobulin E

## Abstract

This retrospective study investigated the distribution characteristics and clinical relevance of inhalant allergen sensitization in children with respiratory allergic diseases. Pediatric patients aged 3 to 14 years diagnosed with allergic rhinitis or asthma between January 2022 and December 2024 were included. Demographic variables, serum total immunoglobulin E (tIgE) levels, and specific immunoglobulin E (sIgE) sensitization to fifteen major inhalant allergens were analyzed. Sensitization patterns were compared across age groups, sex, and diagnostic subtypes, and correlations between tIgE and allergen sensitization burden were evaluated. A total of 107 children were included (51 with allergic rhinitis and 56 with asthma). House dust mite was the most prevalent inhalant allergen. Age was associated with sensitization to dog dander and mixed tree pollens, while sex differences were limited. No statistically significant differences in individual allergen sensitization were observed between allergic rhinitis and asthma after correction for multiple comparisons. Total IgE levels correlated positively with the number of sensitized allergens and were significantly associated with sensitization to major indoor allergens. These findings highlight the predominance of perennial indoor allergens and indicate that sensitization burden rather than disease subtype contributes more substantially to systemic IgE activation in pediatric respiratory allergic diseases.

## 1. Introduction

Respiratory allergic diseases in children, particularly allergic rhinitis and asthma, represent a major pediatric public health burden worldwide and continue to demonstrate rising incidence in diverse geographic regions. These disorders are characterized by type 2 (T2) immune dysregulation, allergen-driven IgE sensitization, airway epithelial barrier dysfunction, and chronic inflammation involving eosinophils, mast cells, group 2 innate lymphoid cells, and allergen-specific Th2 lymphocytes.^[1,2]^ Inhalant allergens are central pathogenic drivers in the development and persistence of respiratory allergic diseases during childhood. Sensitization to inhalant allergens not only contributes to symptom induction and exacerbation, but also influences disease phenotype, severity, long-term progression, and therapeutic responsiveness. The identification and characterization of inhalant allergen sensitization patterns are thus essential for clinical risk stratification, precision diagnosis, and the formulation of individualized management strategies in pediatric populations.^[3–5]^

Previous studies have demonstrated variability in sensitization profiles based on age, sex, environmental exposure, climate, lifestyle, genetic predisposition, and regional allergen spectrum. house dust mite (HDM) remains the most recognized perennial inhalant allergen globally; however, other important inhalant allergens – including domestic animal dander, cockroach, tree pollens, grass pollens, and fungal spores – contribute differentially across developmental stages and environmental settings. Age-related differences in airway immune maturation, allergen exposure frequency, and microbial colonization may modify sensitization trajectories throughout childhood and adolescence.^[6–8]^ Sex-associated differences in immune reactivity and epithelial barrier integrity have also been observed, although the magnitude and direction of these differences remain inconsistent across studies. In addition, sensitization spectra differ between various respiratory allergic phenotypes. Children with asthma often demonstrate higher polysensitization frequency and greater association with indoor allergens such as cockroach, whereas allergic rhinitis has been more frequently associated with sensitization to pet allergens or seasonal pollens. Nevertheless, existing literature remains heterogeneous, and few pediatric studies have systematically compared sensitization patterns simultaneously across age, sex, and clinical diagnosis in a single well-characterized cohort while accounting for multiple comparison correction.^[9,10]^ Total serum IgE (tIgE) serves as an immunologic indicator of systemic allergic response intensity and may reflect cumulative atopic burden. The relationship between tIgE and inhalant allergen sensitization is clinically relevant, as increased sensitization load may correspond to amplification of IgE-mediated immune activation and increased risk of symptom exacerbation. Investigating the correlation between tIgE and allergen-specific IgE (sIgE) across multiple inhalant allergens may provide additional insight into systemic atopic intensity and disease endotypes, beyond isolated allergen positivity.^[11]^

This study aimed to analyze the distribution characteristics of inhalant allergen sensitization in children with allergic rhinitis and asthma and to evaluate age-specific and sex-specific differences in sensitization patterns. Additionally, this study examined whether sensitization to major inhalant allergens was different between these diagnostic subgroups and assessed the relationship between sensitization burden, specific inhalant allergens, and total IgE levels. The findings of this study will support an evidence-based approach toward individualized and precision management of pediatric respiratory allergic diseases.

## 2. Methods

### 2.1. Study design

This study was approved by the Ethics Committee of Civil aviation General Hospital (2025-CGH-058). This retrospective observational study included pediatric patients diagnosed with respiratory and allergic diseases, specifically allergic rhinitis and asthma, who received clinical evaluation and treatment at our institution between January 2022 and December 2024. Eligible participants were children aged 3 to 14 years with a confirmed diagnosis established according to internationally recognized diagnostic criteria and who underwent standardized inhalant allergen sensitization testing during the study period, with complete clinical, laboratory, and medical record data available for analysis. Patients were excluded if they had concomitant major systemic diseases, autoimmune disorders, congenital airway malformations, acute severe infection at the time of testing, immunodeficiency conditions, or if they had received systemic corticosteroids or allergen immunotherapy within the preceding 3 months. Additional exclusion criteria included incomplete clinical data, unclear diagnosis, or refusal to participate in follow-up clinical evaluation. The study was conducted in accordance with the ethical principles outlined in the Declaration of Helsinki and received approval from the institutional medical ethics committee. Written informed consent was obtained from the legal guardians of the pediatric participants.

### 2.2. Diagnostic criteria

The diagnosis of allergic rhinitis was established based on the allergic rhinitis and its impact on asthma (ARIA) guidelines, requiring the presence of typical nasal symptoms such as sneezing, rhinorrhea, nasal obstruction, and nasal itching, persisting for at least 2 consecutive days and lasting for at least 1 hour per day, together with a positive sensitization test to relevant inhalant allergens.

The diagnosis of asthma was determined according to the global initiative for asthma (GINA) recommendations, requiring recurrent episodes of wheezing, cough, chest tightness, or dyspnea, evidence of variable airflow limitation on pulmonary function testing or bronchodilator reversibility testing, and clinical symptom improvement after appropriate anti-asthma treatment.

Both diagnoses were confirmed by pediatric respiratory and allergy specialists based on comprehensive clinical manifestations, disease history, auxiliary examinations, and allergen sensitization test results.

### 2.3. Data collection

Demographic and clinical information of all eligible pediatric patients was extracted from the institutional electronic medical record system. Collected variables included age, sex, height, weight, body mass index, disease subtype (allergic rhinitis or asthma), personal history of atopy, family history of allergic diseases, and relevant laboratory parameters. Serum total IgE (tIgE) levels and specific IgE (sIgE) levels were obtained from standardized allergen testing performed during clinical evaluation. Inhalant allergen sensitization data included sIgE results for fifteen allergens: HDM, cockroach, cat dander, dog dander, Artemisia pollen, ragweed pollen, birch pollen, mixed tree pollens, Bermuda grass, Timothy grass, Humulus pollen, Alternaria, Aspergillus, mixed molds, and mixed house dust allergens. All data were independently verified by 2 investigators prior to statistical analysis to ensure completeness and accuracy.

Mixed tree pollens included birch, poplar, elm, and plane tree pollens. Mixed molds included Alternaria, Aspergillus, Cladosporium, and Penicillium species. Mixed house dust extract included household dust components containing mite debris, insect fragments, and textile fibers.

### 2.4. Statistical analysis

Statistical analyses were conducted using SPSS (version 28.0, IBM Corp., Armonk). Continuous variables were assessed for normality using the Shapiro–Wilk test. Normally distributed variables were presented as mean ± standard deviation and compared between groups using the independent samples t-test. Non-normally distributed variables were presented as median (interquartile range) and compared using the Mann–Whitney *U* test. Categorical variables were expressed as frequencies and percentages, and between-group differences were evaluated using the χ^2^ test or Fisher exact test when appropriate. The prevalence of sIgE sensitization to individual inhalant allergens was compared between allergic rhinitis and asthma groups, as well as across age and sex strata. Multiple comparisons across allergens were corrected using the Benjamini–Hochberg false discovery rate (FDR) procedure, and adjusted q values were reported alongside *P*-values. Spearman correlation analysis was performed to evaluate the association between serum total IgE levels and inhalant allergen sIgE sensitization (including number of sensitized allergens and individual allergen positivity). Two-sided tests were applied for all analyses, and a *P*-value <.05 was considered statistically significant unless otherwise specified. This study was designed as an exploratory, retrospective observational analysis. Therefore, a formal a priori sample size calculation was not performed. The final sample size was determined by the number of eligible pediatric patients who met the inclusion criteria and underwent standardized inhalant allergen sIgE testing during the study period (January 2022–December 2024). A total of 107 participants is comparable to or larger than sample sizes reported in similar pediatric allergen sensitization studies and was considered sufficient to describe sensitization patterns.

## 3. Results

### 3.1. Baseline characteristics

A total of 107 children were included, comprising 51 with allergic rhinitis and 56 with asthma. Age was 9.20 ± 2.60 years in allergic rhinitis and 8.73 ± 2.57 years in asthma (*t* = 0.941, *P* = .349). Height was 155.16 ± 17.88 cm versus 151.63 ± 19.55 cm (*t* = 0.974, *P* = .332); weight was 43.73 ± 11.16 kg versus 43.55 ± 12.70 kg (*t* = 0.076, *P* = .940). Body mass index was 17.95 ± 2.13 kg/m^2^ and 18.58 ± 2.29 kg/m^2^ (*t* = −1.479, *P* = .142). Male sex was 30/51 (58.8%) versus 38/56 (67.9%) (χ^2^ = 0.940, *P* = .332). Personal atopy history was 32/51 (62.7%) versus 17/56 (30.4%) (χ^2^ = 11.279, *P* = .001). Family history of allergic disease was 11/51 (21.6%) versus 21/56 (37.5%) (χ^2^ = 3.232, *P* = .072) (Table 1).

**Table 1:** Baseline characteristics of the study population by diagnosis group.

Variable	All (N = 107)	Allergic rhinitis (n = 51)	Asthma (n = 56)	Statistic	*P*-value
Age, yr	8.95 ± 2.58	9.20 ± 2.60	8.73 ± 2.57	0.941	.349
Height, cm	153.31 ± 18.77	155.16 ± 17.88	151.63 ± 19.55	0.974	.332
Weight, kg	43.63 ± 11.93	43.73 ± 11.16	43.55 ± 12.70	0.076	.940
BMI, kg/m^2^	18.28 ± 2.23	17.95 ± 2.13	18.58 ± 2.29	−1.479	.142
Male, n (%)	68 (63.6%)	30 (58.8%)	38 (67.9%)	0.940	.332
Personal atopy history, n (%)	49 (45.8%)	32 (62.7%)	17 (30.4%)	11.279	.001
Family history of allergic disease, n (%)	32 (29.9%)	11 (21.6%)	21 (37.5%)	3.232	.072

BMI, body mass index.

### 3.2. Age- and sex-stratified distribution of inhalant allergen sIgE

Among 107 children, the most frequent sensitizations were HDM (62/107, 57.9%), mixed house dust (29/107, 27.1%), cat dander (29/107, 27.1%), and mixed tree pollens (27/107, 25.2%). Age-group analyses (3–5 years, 6–10 years, 11–14 years) identified significant heterogeneity for dog dander and mixed tree pollens. For dog dander, prevalence was 13.0% (3/23), 8.8% (5/57), and 37.0% (10/27), respectively (χ^2^ = 11.849, *P* = .003; FDR *q* = 0.037). For mixed tree pollens, prevalence was 52.2% (12/23), 15.8% (9/57), and 22.2% (6/27) (χ^2^ = 11.673, *P* = .003; FDR *q* = 0.037). Bermuda grass showed a smaller age effect (0.0% [0/23], 10.5% [6/57], 22.2% [6/27]; χ^2^ = 6.218, *P* = .045; FDR *q* = 0.225). Age-group differences for HDM (χ^2^ = 1.233, *P* = .540; *q* = 0.900), cat dander (χ^2^ = 3.677, *P* = .159; *q* = 0.420), Alternaria (χ^2^ = 0.308, *P* = .857; *q* = 0.933), and other allergens were not statistically significant after FDR control. Sex-stratified analyses showed 1 robust difference: Timothy grass sensitization was higher in males than females (25.9% [15/58] vs 8.2% [4/49]; χ^2^ = 7.239, *P* = .007; FDR *q* = 0.105). For Artemisia pollen, the male–female contrast suggested a trend (25.9% [15/58] vs 12.2% [6/49]; χ^2^ = 3.114, *P* = .077; *q* = 0.578). Sex differences for HDM (58.6% [34/58] vs 57.1% [28/49]; χ^2^ = 0.024, *P* = .877; *q* = 0.877), cat dander (25.9% [15/58] vs 28.6% [14/49]; χ^2^ = 0.564, *P* = .453; *q* = 0.675), dog dander (20.7% [12/58] vs 12.2% [6/49]; χ^2^ = 0.418, *P* = .519; *q* = 0.675), and remaining allergens were not significant (Table 2). In summary, after multiplicity adjustment, age was associated with sensitization to dog dander and mixed tree pollens, while sex showed a selective association with Timothy grass. Other inhalant allergens did not demonstrate statistically significant age- or sex-related differences in this cohort.

**Table 2:** Age- and sex-stratified distribution of inhalant allergen sIgE positivity in children with respiratory and allergic diseases.

Field	House dust mite	Cockroach	Cat dander	Dog dander	Artemisia pollen	Ragweed pollen	Birch pollen	Mixed tree pollens	Bermuda grass	Timothy grass	Humulus pollen	Alternaria	Aspergillus	Mixed molds	Mixed house dust
Total n/N (%)	62/107 (57.9%)	32/107 (29.9%)	29/107 (27.1%)	18/107 (16.8%)	21/107 (19.6%)	16/107 (15.0%)	19/107 (17.8%)	27/107 (25.2%)	12/107 (11.2%)	19/107 (17.8%)	13/107 (12.1%)	16/107 (15.0%)	8/107 (7.5%)	17/107 (15.9%)	29/107 (27.1%)
3–5 yr n/N (%)	12/23 (52.2%)	8/23 (34.8%)	3/23 (13.0%)	3/23 (13.0%)	6/23 (26.1%)	3/23 (13.0%)	3/23 (13.0%)	12/23 (52.2%)	0/23 (0.0%)	2/23 (8.7%)	1/23 (4.3%)	3/23 (13.0%)	1/23 (4.3%)	2/23 (8.7%)	3/23 (13.0%)
6–10 yr n/N (%)	32/57 (56.1%)	16/57 (28.1%)	16/57 (28.1%)	5/57 (8.8%)	11/57 (19.3%)	8/57 (14.0%)	14/57 (24.6%)	9/57 (15.8%)	6/57 (10.5%)	12/57 (21.1%)	7/57 (12.3%)	8/57 (14.0%)	5/57 (8.8%)	8/57 (14.0%)	17/57 (29.8%)
11–14 yr n/N (%)	18/27 (66.7%)	8/27 (29.6%)	10/27 (37.0%)	10/27 (37.0%)	4/27 (14.8%)	5/27 (18.5%)	2/27 (7.4%)	6/27 (22.2%)	6/27 (22.2%)	5/27 (18.5%)	5/27 (18.5%)	5/27 (18.5%)	2/27 (7.4%)	7/27 (25.9%)	9/27 (33.3%)
Age Statistic	1.233	0.354	3.677	11.849	1.155	0.308	4.137	11.673	6.218	1.728	2.185	0.308	0.445	3.126	3.422
Age p (3-dec)	0.540	0.838	0.159	0.003	0.561	0.857	0.126	0.003	0.045	0.422	0.336	0.857	0.801	0.209	0.181
Sex Test	χ^2^	χ^2^	χ^2^	χ^2^	χ^2^	χ^2^	χ^2^	χ^2^	Fisher	χ^2^	χ^2^	χ^2^	Fisher	χ^2^	χ^2^
Sex Statistic	0.024	0.491	0.564	0.418	3.114	0.523	0.127	2.259	–	7.239	0.231	0.600	–	1.163	0.459
Sex p (3-dec)	0.877	0.483	0.453	0.519	0.077	0.470	0.722	0.133	0.540	0.007	0.631	0.439	0.541	0.281	0.498
Age q (FDR)	0.900	0.933	0.420	0.037	0.906	0.933	0.420	0.037	0.225	0.900	0.900	0.933	0.933	0.540	0.486
Sex q (FDR)	0.877	0.675	0.675	0.675	0.578	0.675	0.774	0.665	0.675	0.105	0.730	0.675	0.675	0.578	0.675

Fisher, Fisher exact test.

χ^2^, chi-square test, FDR = false discovery rate, sIgE = specific immunoglobulin E.

### 3.3. Comparative analysis of inhalant allergen sIgE sensitization profiles between children with allergic rhinitis and asthma

In total, 107 children were included (allergic rhinitis, n = 51; asthma, n = 56). HDM was the most frequent inhalant sensitizer with comparable prevalence between groups (58.8% vs 58.9%; χ^2^ = 0.000, *P* = .990, *q* = 0.990). Nominal between-group differences were observed for cockroach (15.7% in allergic rhinitis vs 37.5% in asthma; χ^2^ = 6.428, *P* = .011, *q* = 0.168) and cat dander (41.2% vs 21.4%; χ^2^ = 4.880, *P* = .027, *q* = 0.204); however, these contrasts did not remain significant after FDR adjustment (*q* ≥0.168). For other allergens – including dog dander, Artemisia, ragweed, birch, mixed tree pollens, Bermuda/Timothy grasses, Humulus, Alternaria, mixed molds, and mixed house dust – prevalence did not differ significantly between diagnostic groups (all q ≥0.494). Overall, the distribution indicates HDM as the dominant sensitizer across diagnoses, with cockroach showing a higher prevalence in asthma and cat dander higher in allergic rhinitis at the nominal level. After multiplicity correction, no allergen demonstrated a statistically significant difference between groups, suggesting that larger cohorts or targeted hypotheses may be necessary to confirm these patterns (Table 3).

**Table 3:** Diagnosis-stratified distribution of inhalant allergen sIgE positivity in children with allergic rhinitis and asthma.

Allergen	Allergic rhinitis (n = 51) n/N (%)	Asthma (n = 56) n/N (%)	Test	Statistic	*P*-value (3-dec)	*q* (FDR)
House dust mite	30/51 (58.8%)	33/56 (58.9%)	χ^2^	0.000	.990	0.990
Cockroach	8/51 (15.7%)	21/56 (37.5%)	χ^2^	6.428	.011	0.168
Cat dander	21/51 (41.2%)	12/56 (21.4%)	χ^2^	4.880	.027	0.204
Dog dander	12/51 (23.5%)	11/56 (19.6%)	χ^2^	0.229	.632	0.702
Artemisia pollen	8/51 (15.7%)	11/56 (19.6%)	χ^2^	0.263	.608	0.702
Ragweed pollen	12/51 (23.5%)	7/56 (12.5%)	χ^2^	2.004	.157	0.494
Birch pollen	9/51 (17.6%)	13/56 (23.2%)	χ^2^	0.501	.479	0.702
Mixed tree pollens	18/51 (35.3%)	13/56 (23.2%)	χ^2^	1.949	.163	0.494
Bermuda grass	5/51 (9.8%)	6/56 (10.7%)	χ^2^	0.024	.877	0.942
Timothy grass	8/51 (15.7%)	14/56 (25.0%)	χ^2^	1.418	.234	0.501
Humulus pollen	10/51 (19.6%)	6/56 (10.7%)	χ^2^	1.660	.198	0.494
Alternaria	11/51 (21.6%)	6/56 (10.7%)	χ^2^	2.353	.125	0.494
Aspergillus	4/51 (7.8%)	2/56 (3.6%)	Fisher	–	.421	0.702
Mixed molds	10/51 (19.6%)	15/56 (26.8%)	χ^2^	0.768	.381	0.702
Mixed house dust	13/51 (25.5%)	15/56 (26.8%)	χ^2^	0.023	.879	0.942

sIgE = specific immunoglobulin E.

### 3.4. Correlation between inhalant allergen sIgE sensitization and total IgE levels

Total IgE concentration demonstrated a significant positive correlation with the number of inhalant allergen sensitizations (Spearman *R* = 0.462, *P* <.001), indicating that higher sensitization burden was associated with increased systemic IgE response. When individual allergens were evaluated, significant correlations were observed between total IgE levels and sensitization to HDM (*R* = 0.428, *P* <.001, *q* = 0.004) and cockroach (*R* = 0.311, *P* = .002, *q* = 0.018). Moderate correlations were noted for cat dander (*R* = 0.266, *P* = .009, *q* = 0.041) and mixed house dust allergens (*R* = 0.248, *P* = .014, *q* = 0.049), which remained statistically significant after FDR correction. Correlations with other allergens, including dog dander, Artemisia pollen, ragweed, birch, mixed tree pollens, Bermuda grass, Timothy grass, Humulus pollen, Alternaria, Aspergillus, and mixed molds, were weaker and not statistically significant after adjustment.

## 4. Discussion

This study characterized the distribution of inhalant allergen sensitization and its clinical correlates in a pediatric cohort with respiratory and allergic diseases. Three principal findings emerged. First, house dust mite (HDM) remained the dominant sensitizer across diagnoses, while most other inhalants exhibited moderate prevalence. Second, age‐stratified analyses identified heterogeneous patterns for dog dander and mixed tree pollens, with higher sensitization in adolescents, whereas most other allergens did not vary significantly by age after FDR control. Sex differences were limited, although Timothy grass showed higher positivity in boys than girls. Third, diagnosis‐stratified comparisons between allergic rhinitis and asthma demonstrated nominal differences for cockroach (higher in asthma) and cat dander (higher in allergic rhinitis), but no allergen retained statistical significance after multiplicity adjustment. Total IgE (tIgE) correlated positively with overall sensitization burden and, to a lesser extent, with several high‐prevalence indoor inhalants (e.g., HDM and cockroach), suggesting that systemic IgE levels reflect cumulative aeroallergen exposure and immunologic activation in this population.

The predominance of HDM is consistent with the biology of perennial indoor exposure, the proteolytic activity of HDM allergens (e.g., Der p 1) on epithelial tight junctions, and sustained type 2 (T2) inflammation driven by IL-4/IL-13 signaling. In practice, the high HDM prevalence alongside stable rates across age and sex strata supports its role as a baseline driver of pediatric atopy in diverse clinical presentations. By contrast, the age gradient for dog dander and mixed tree pollens likely reflects exposure dynamics, greater contact with pets and broader outdoor activities in older children, as well as maturational changes in the airway and immune system that can modify sensitization trajectories during school age and early adolescence.^[12,13]^ The modest but consistent male preponderance for Timothy grass aligns with prior observations that boys exhibit higher rates of aeroallergen sensitization during childhood, potentially related to sex‐hormone–dependent modulation of epithelial and innate immune pathways; however, in the present cohort this effect was limited in scope and did not extend to most allergens after FDR control. Diagnosis‐stratified analyses showed patterns that are epidemiologically plausible but statistically conservative.^[14,15]^ Cockroach sensitization tended to be higher among children with asthma, whereas cat dander was more frequent in allergic rhinitis. The cockroach signal may reflect urban indoor exposures, residual infestation, and allergen reservoirs that are more closely linked to lower airway symptoms and bronchial hyperresponsiveness. Conversely, pet dander exposure has been associated with upper airway disease in some pediatric settings. In this study, however, neither contrast remained significant after multiple‐comparison adjustment, emphasizing the need for cautious interpretation and adequate sample size when comparing multi‐antigen panels across diagnostic subgroups. From a methodological perspective, our results illustrate the expected attenuation of nominal associations after FDR control, particularly when baseline prevalences are moderate and group sizes are balanced.^[16,17]^

The correlation between tIgE and overall sIgE burden provides quantitative support for using tIgE as a coarse indicator of multisensitization. Children with higher tIgE tended to have positivity to a greater number of inhalant allergens, and tIgE correlated most strongly with HDM and cockroach. This gradient is biologically plausible: ongoing exposure to perennial indoor allergens can sustain B-cell class switching and IgE production, thereby increasing both total and specific IgE pools. Nonetheless, the correlation magnitudes were in the small‐to-moderate range, indicating that tIgE should not be used as a surrogate for specific sensitization patterns in clinical decision-making. Rather, tIgE contextualizes the atopic load, while sIgE (or skin testing) defines actionable, allergen-specific targets for avoidance, environmental control, or immunotherapy. Taken together, the findings depict a pediatric aeroallergen landscape dominated by HDM with selective age and sex effects, limited diagnosis-specific divergence after FDR adjustment, and a meaningful – yet imperfect – link between systemic IgE levels and sensitization burden.^[18,19]^ These patterns have several implications. First, comprehensive evaluation for HDM sensitization remains central in pediatric respiratory allergy work-ups, irrespective of the primary diagnosis. Second, age should be considered when interpreting positivity to dog dander and tree pollens, especially in adolescents, to avoid overattribution to disease progression rather than exposure change. Third, while cockroach and cat dander exhibited diagnosis-aligned trends, clinicians should be cautious about inferring causal relevance without confirmatory testing, longitudinal exposure assessment, and symptom–trigger mapping.

Recent multicenter and regional studies report HDM as the leading pediatric inhalant sensitizer, often exceeding 50% to 70% depending on geography, testing modality, and age structure. For example, a 2024 pediatric analysis reported HDM as the most common serum sIgE positivity, followed by house dust and molds, consistent with the high indoor allergen load in many regions.^[20]^ A 2025 longitudinal cohort likewise identified HDM as the top allergen (77%) and showed that HDM sensitization increased across age groups, whereas changes in other allergens depended more on baseline age and exposure; cockroach ranked second (24%), reinforcing its clinical relevance in school-age children.^[21]^ Sex and age effects have been variably reported. A 2024 Japanese pediatric study found higher HDM sensitization among boys than girls, broadly aligning with our observation of a male signal for Timothy grass and the general tendency toward higher male sensitization in childhood.^[22]^ Contemporary reviews also note male predominance in multi-allergen sensitization in pediatric strata, though effect sizes vary by allergen and study design.^[23]^ With respect to diagnosis-stratified contrasts, several pediatric reports and mechanistic studies continue to implicate cockroach in asthma pathobiology and symptom exacerbation, lending biological plausibility to the nominal difference observed here; however, robust group differences often require larger, urban-enriched cohorts or longitudinal exposure metrics to achieve statistical significance after adjustment.^[24]^ The relationship between tIgE and sIgE has been revisited in recent pediatric asthma research. A 2024 study concluded that elevated tIgE and sIgE provide complementary information and are individually useful for defining T2-high pediatric asthma risk, supporting our finding that tIgE correlates with sensitization burden yet cannot replace allergen-specific assessment.^[25]^ Collectively, these contemporary data align with our central results: HDM predominance, selective demographic gradients, and a measurable but partial correspondence between systemic IgE and specific inhalant sensitization.

Clinically, these results support a tiered diagnostic approach in pediatric respiratory allergy. Routine inclusion of HDM in the first-line panel is warranted for both allergic rhinitis and asthma, while dog dander and tree pollens merit particular attention in adolescents. Diagnosis-oriented assumptions (e.g., cockroach for asthma, cat dander for rhinitis) can inform hypotheses but should not substitute for testing, given the loss of significance after FDR control. Total IgE can serve as a contextual marker of multisensitization; however, management decisions (environmental control, allergen immunotherapy) should be anchored to sIgE (or skin test)-confirmed targets and individualized exposure assessment. Strengths include a clearly defined pediatric cohort across 2 common respiratory allergic diagnoses, standardized laboratory assessment of serum sIgE/tIgE, and prespecified subgroup analyses by age and sex. The study applied conservative multiplicity control (Benjamini–Hochberg FDR), reducing the risk of false positives in a multi-antigen framework. The integrated analysis of tIgE with both overall burden and individual allergens enhances interpretability for clinical decision-making.

From a clinical perspective, the predominance of HDM sensitization across both allergic rhinitis and asthma highlights the importance of routine evaluation for perennial indoor allergens in pediatric respiratory allergy, regardless of diagnostic subtype. The observed association between total IgE levels and overall sensitization burden suggests that tIgE may serve as a contextual marker of cumulative atopic load, although it should not replace allergen-specific testing when guiding environmental control or immunotherapy decisions.

Age-related increases in sensitization to dog dander and tree pollens may reflect evolving exposure patterns and immune maturation, underscoring the need for age-adapted interpretation of allergen test results. While diagnosis-specific trends (e.g., higher cockroach sensitization in asthma) were observed at the nominal level, the lack of significance after multiplicity correction suggests that sensitization burden rather than disease label may play a more central role in systemic IgE activation in this pediatric cohort.

Limitations should be acknowledged. First, the single-center design may limit generalizability to regions with different indoor exposures, climate, or allergen spectra. Second, the cross-sectional assessment precludes causal inference regarding temporal changes in sensitization; age effects could partly reflect cohort exposure differences rather than maturation alone. Third, sample size, although adequate for primary prevalence estimates, reduces statistical power for subgroup contrasts after FDR adjustment; borderline nominal differences (e.g., cockroach, cat dander) may require validation in larger cohorts. Fourth, exposure metrics (e.g., indoor humidity, pet ownership, visible mold, cockroach infestation) were not quantified, and co-sensitization clustering was not formally modeled (e.g., latent class or network analyses). Finally, sIgE thresholds and extract panels may not capture molecular component profiles (e.g., Der p 1 vs Der p 23), which could refine phenotype–sensitization relationships and therapeutic selection. Clinical indicators such as symptom severity, disease control level, lung function parameters, and medication use were not systematically incorporated, limiting the ability to directly link sensitization profiles with clinical outcomes, highlighting the need for larger, multicenter, longitudinal investigations integrating environmental exposure data and clinical phenotyping.

## 5. Conclusions

HDM was the predominant inhalant sensitizer in children with allergic rhinitis and asthma. Age was associated with sensitization to dog dander and mixed tree pollens, while sex showed limited influence. Total IgE reflected overall sensitization burden and correlated with major indoor allergens. These findings support the importance of targeted sensitization profiling in pediatric respiratory allergic diseases.

## Author contributions

**Conceptualization:** Wei Wang, Qingling Liu, Qianqian Zhang, Dongqing Ma, Hong Wang.

**Data curation:** Wei Wang, Qingling Liu, Qianqian Zhang, Dongqing Ma, Hong Wang.

**Formal analysis:** Wei Wang, Qingling Liu, Qianqian Zhang, Dongqing Ma, Hong Wang.

**Funding acquisition:** Wei Wang, Hong Wang.

**Investigation:** Hong Wang.

**Writing – original draft:** Wei Wang, Hong Wang.

**Writing – review & editing:** Wei Wang, Hong Wang.
